# Experiences of perceived vs actual time required for data management on electronic data capture clinical trials in a non commercial setting

**DOI:** 10.1186/1745-6215-14-S1-O13

**Published:** 2013-11-29

**Authors:** Nancy Tappenden, Lindsey Masters

**Affiliations:** 1 MRC Clinical Trials Unit Hub for Trials Methodology Research, London, UK

## 

The traditional method of data management at the MRC Clinical Trials Unit has been for paper CRFs to be completed by accredited sites and sent in to a coordinating data centre. There the data is entered on to a trial database and cleaned. However there has been a shift recently within our unit towards electronic data capture, whereby sites enter data directly into the trial database. Data monitoring is done at the coordinating centre and cleaning occurs at both the coordinating centre and site.

The perception is that this method provides better data quality and essentially reduces the amount of time needed for data management at the co-ordinating data centre. Experience so far has shown that this may not be the case. The time required for data management is in fact directed toward less common tasks such as training and ongoing assistance to site staff in using the trial database. It appears that there is increased resource required for the database programmers due to novel data capture methodologies. There also seems to be an increase in the time spent at sites on collecting and entering the data, especially if, due to clinic conditions, they are completing paper CRFs first before data entry.

In this presentation, we shall explore the data management activities being undertaken in these trials and compare them to a similar trial employing the more traditional model described above.

